# 

**Published:** 1890-09

**Authors:** 


VOL. XI. SEPTEMBER, 1890. No. 9.

THE

### International Dental Journal

A MONTHLY PERIODICAL

DEVOTED TO

### DENTAL AND ORAL SCIENCE.

**JAMES TRUMAN, D.D.S., Editor.**

Publication Office,

716 FILBERT STREET, PHILADELPHIA.

PUBLISHED BY THE

INTERNATIONAL DENTAL PUBLICATION COMPANY,

51 WEST THIRTY-SEVENTH STREET, NEW YORK CITY,

—AND—

716 FILBERT STREET, PHILADELPHIA.

ENTERED AT PHILADELPHIA POST-OFFICE AS SECOND-CLASS READING MATTER.

$2.50 per Annum, in Advance. Single Copies, 25 Cents.

PRINTED BY J. B. LIPPINCOTT COMPANY, PHILADELPHIA.

